# Mud Volcanoes of Trinidad as Astrobiological Analogs for Martian Environments

**DOI:** 10.3390/life4040566

**Published:** 2014-10-13

**Authors:** Riad Hosein, Shirin Haque, Denise M. Beckles

**Affiliations:** 1 Department of Chemistry, University of the West Indies, St. Augustine, Trinidad, West Indies, Trinidad and Tobago; E-Mails: riadhosein83@gmail.com (R.H.); denise.beckles@sta.uwi.edu (D.M.B.); 2 Department of Physics, University of the West Indies, St. Augustine, Trinidad, West Indies, Trinidad and Tobago

**Keywords:** mud volcanoes, Trinidad, chemical profiling, microbial life, analog, Mars

## Abstract

Eleven onshore mud volcanoes in the southern region of Trinidad have been studied as analog habitats for possible microbial life on Mars. The profiles of the 11 mud volcanoes are presented in terms of their physical, chemical, mineralogical, and soil properties. The mud volcanoes sampled all emitted methane gas consistently at 3% volume. The average pH for the mud volcanic soil was 7.98. The average Cation Exchange Capacity (CEC) was found to be 2.16 kg/mol, and the average Percentage Water Content was 34.5%. Samples from three of the volcanoes, (i) Digity; (ii) Piparo and (iii) Devil’s Woodyard were used to culture bacterial colonies under anaerobic conditions indicating possible presence of methanogenic microorganisms. The Trinidad mud volcanoes can serve as analogs for the Martian environment due to similar geological features found extensively on Mars in Acidalia Planitia and the Arabia Terra region.

## 1. Introduction

Mars is one of the key places of interest in the solar system for the possible detection of microbial life [1]. The conditions on Mars are not totally dissimilar to those on Earth, due to their relative proximity to each other. The harsh Martian climate and geography is comparable to some places on Earth and geological features like mud volcanoes are common to both [2]. Mud volcanism was found to be very pervasive in Acidalia Planitia on Mars. Oehler and Allen mapped more than 18,000 circular mounds representative of mud volcanism and estimate that more than 40,000 of these features could exist in the region [2]. The presence of such mud volcanoes on Mars have been reported in the regions of Utopia, Isidis, Northern Borealis and Scandia from several studies as early as 1995 [3]. The Firsoff crater in the Arabia Terra region possesses hundreds of mound-like features interpreted as mud volcanoes as well [4]. Mud volcanism is the closest analog in terrestrial geology to these Martian features [5]. Similar to mud volcanoes on Earth, Martian mud volcanoes would have transported materials from the depths to the surface providing possible signatures of past life on Mars if there was such. Some of the best studied terrestrial mud volcanoes are in Azerbaijan and the Caspian Sea [6], however the onshore mud volcanoes in the Caribbean region are not well known and studied from a Martian analog perspective.

Morphological evidence such as river beds and surface volcanism show that Mars possesses similar geological structures to Earth, and also was once just as active [7]. It is therefore speculated that extremophiles may have evolved independently on Mars along similar terrestrial conditions and constraints as life did on Earth.

The detection of water molecules on Mars contributes significant evidence to the argument that Mars could have once sustained life in the past [8]. Differences in the atmospheric conditions between Mars and Earth exist, but the similarities between the geology of the two planets make for a plausible argument that microbial life could have potentially existed on Mars in the past. Detection of methane gas (CH_4_) on a planet or moon can be a possible signature of life as a metabolic byproduct of microbial life as happens on Earth. However, there may be a geochemical explanation for the origin of the methane from the mud volcanoes as well. On Earth, the majority of the methane budget is produced by biogenic sources, in wet conditions of anoxic environments and if there is conclusive evidence that there is methane on Mars, then it could be coming in part from a biological activity in subsurface Mars [9].

While methane (CH_4_) was discovered in localized regions on Mars from several reports at the 10–60 ppbv level [10], and methane has been identified in the two main volcano provinces of Tharsis and Elysium [11,12], its detection is not without controversy. The discovery of methane in 2004 was concentrated in the areas above the mud volcanoes with evidence showing that these volcanoes may still be producing methane that is present in the Martian atmosphere [13]. However, the actual sources of the gas remain unknown [14]. Zahnle *et al.* [15] argue that the scientific robustness of the spatial and temporal variability reported is inconsistent with Mars’s atmospheric chemistry. More recent findings from *in situ* measurements at Gale Crater by Curiosity rover put an upper limit at only 1.3 ppbv [16]. The Tunable Laser Spectrometer (TLS) aboard Curiosity used for this detection is superior to the previous ground based and orbiting spectrometers and thus there is a serious controversy on the presence and variability of methane on Mars. The Indian Mars Orbiter Mission (MOM) and NASA’s Mars Atmosphere and Volatile EvolutioN (MAVEN), both scheduled for entry into the Martian atmosphere in September 2014 should help to shed further light on the methane controversy.

Although the issue of methane on Mars is far from settled, it is still important to consider the mineralogy of the terrestrial mud volcanoes due to the similar geological structures on Mars. This is particularly so in light of the findings from Curiosity of the fluvio-Lacustrine environment at Yellowknife Bay, Gale Crater [17]. The Mars Science Laboratory (MSL) rover Curiosity also searched for habitable environments for microorganisms based on the geochemistry [18]. Understanding the terrestrial biology at the mud volcanoes serves as an analog for such studies. Possible terrestrial-like methanogens living on subsurface Mars may share unique efficient metabolic strategies geared towards harvesting the limited nutrients in their environment.

Nonetheless, the possibility of methane on Mars combined with similar geological features of mud volcanoes, on Mars is significant reason to warrant the study of mud volcanoes as analog to the Martian environment. The mud volcanoes of Trinidad in our study are geologically active while the mud volcanoes on Mars are presently extinct. However, in the geological history of Mars they would have been active at some time to produce the morphology that is observed today [19].

Methane gas is one of the component gases being emitted from terrestrial mud volcanoes, and it is responsible for a significant contribution to the global methane budget of Earth [20]. The renewable methane on Earth is a metabolic by-product of terrestrial microorganisms, predominantly methanogens such as methanogenic Archaea. Therefore, terrestrial mud volcanoes can be used as an analog to help identify variables essential in supporting resident volcano microbes and under similar constraints to explain the possible existence of life on subsurface Mars [21].

The Martian surface cannot support life, as the atmosphere is continually bombarded by adverse environmental stressors, such as solar radiation, temperature, pressure, radiation and harmful anti-organic chemical molecules [22]. Additionally, the lack of liquid water also contributes to the non-proliferation of life on the surface. It is therefore assumed that if there is microbial life, then it exists in volcano reservoirs in independent volcanic systems beneath the subsurface of Mars.

The subsurface of the Trinidad mud volcanoes are hostile environments for microorganisms with no light and limited nutrients. Such extremophile microorganisms that exist within the mud volcano would do so anaerobically. A study by Ali *et al*. on three of the Trinidad mud volcanoes, Digity, Piparo and Devil’s Woodyard showed the presence of microorganisms existing under anaerobic, aerobic, methane aerobic and methane anaerobic conditions in the mud volcanoes at depths of 0.1 m and 1.0 m [23]. Methanogens found in the mud within the mud volcanoes of Trinidad, use mud volcanic soil constituents as energy sources to support their proliferation, producing methane gas as a metabolic by-product [24]. Furthermore, fossils from the Digity mud volcano were dated as 15 million years old, evidence that the slurry of the mud volcano serves to elucidate geological history of these structures on Earth and so too on Mars.

## 2. Mud Volcanoes and Soil Science

The Trinidad—Eastern Venezuelan Basin is a mobile shale basin with a complex tectonic and stratigraphic history [25]. Mud volcanoes developed along the axis as the basin evolved. These volcanoes are not generally associated with the presence of hydrocarbon accumulations, although a few are present within the oil fields. In the southern region of Trinidad, there is also the largest pitch (hydrocarbon) lake in the world and this has been the site for several astrobiological studies as an analog to hydrocarbon lakes on Titan [26,27]. Studies on the mud volcanoes of Trinidad date from as early as 1911 but have primarily concentrated on mineral content and physical properties [28,29].

The slurry from the mud volcanoes is the anaerobic extremophiles’ immediate environment. Despite the observed variances, conservative features in the abundances and isotopic offsets show microbial biomarkers occur, likely reflecting the overall relationships between Archaea and bacteria and the nature of carbon flow between them [30]. Adaptation is a prerequisite for microbes to take advantage of their natural environment. The study of the interaction between microbe and environment can lead to an understanding of its biology. Extremophile biology must be studied along with the different physical and chemical properties of the soil. The chemical properties of the soil preliminarily investigated were the cation exchange capacity (CEC), pH, the percentage water content, elemental and mineralogical distribution.

Profiling the environment which is the habitat of microbial life gives insight into what energy sources are available to microbes for harvesting and possible mechanisms by which they feed. The soil properties of the mud provide insight on the mineral weathering and the nutrient composition and complex soil interactions between cations present in the soil. For example, phosphorus becomes chemically immobile outside the pH range of 6.0 to 7.0 and many nutrient cations such as zinc (Zn^2+^), aluminum (Al^3+^), iron (Fe^2+^), copper (Cu^2+^), cobalt (Co^2+^), and manganese (Mn^2+^) are soluble and available for uptake by plants below pH 5.0.

### 2.1. Cation Exchange Capacity (CEC)

The clay of any particular soil is generally made up of a mixture of colloidal minerals. The CEC of the soil is determined by the relative amounts of different colloids in that soil and by the CEC of the individual colloids. CEC is influenced by pH. If the CEC is low then the pH of the soil is low. The specific exchangeable cations associated with soil colloids differ from one climatic condition to another and the cations that dominate the exchange complex have a marked influence on soil properties.

CEC is useful in identifying the relative proportions of sources of acidity and alkalinity in the solution. Exchangeable cations generally are available to microorganisms. Cation exchange is the process where hydrogen ions from microorganisms, replace nutrient cations from the exchange complex. The nutrient cations are forced into the soil solution where they can be assimilated by the absorptive surfaces of soil organisms, or they may be removed by drainage water [31].

### 2.2. Soil pH

All colloids, organic or inorganic, exhibit the surface charges associated with OH- groups, and are largely pH dependent and pH affects complex soil interactions. At certain pH’s, nutrients can be rendered chemically immobile for the uptake of nutrients such as cations in the soil such as zinc, cobalt and aluminum [32].

Clay soils are silicate based which constitute the bulk structure. These essential silicates provide housing for terrestrial microorganisms to perform metabolic activities. Mud volcanoes of Trinidad consist mainly of silicate matrices such as kaolinite, montromollinite and vermiculite [33]. Clay minerals have fine particles and high bulk density. High bulk density in clays causes high water retention due to limited porosity, compared to other soil types.

### 2.3. Water Content

Maximum retentive capacity is when all the soil pores are filled with water and the soil is water saturated. Volumetric water content is essentially the same as the total porosity. Water in the largest pores will drain downward quite rapidly when rain or irrigation ceases, after 1–3 days rapid downward movement occurs and becomes negligible thereafter. The soil is now at its “field capacity.” Water acts as a medium of transport; transporting minerals and dissolved chemical constituents in a soil solution. Water is a prerequisite for the many complex soil reactions necessary for microbes to thrive. As such, the water content of the mud volcanoes is an important criterion in order to understand its ability to harbor microbial life.

### 2.4. Mud Volcanism

The expulsion of the mud volcanic ejecta takes place through the process of advection. Fluid advection through the sediments provides an efficient mechanism for the upward transport of reactive components and trace gases, where methane is one of the most important and has an impact on the mineralization within the shallow sediments and on the chemistry and benthic biota [34].

The chemistry and physics of the mud volcanoes in Trinidad were not studied well in the past and although surface features, sediments and gas from the Trinidad mud volcanoes have been studied by several authors, expelled fluids have been mostly neglected [35]. The chemical analyses of the soil of the mud volcano in the past have taken the form of mineralogical evaluation. The Devil’s Woodyard was used as a test site for mud volcano mineralogy. Differential Thermal Analysis (DTA) was done and the results yielded were the composition of 56% SiO_2_, 18% Al_2_O_3_, and 7% Fe_2_O_3_ as the major oxides [33]. Other than Devil’s Woodyard, there have been no other mineralogical evaluations of the different mud volcanoes of Trinidad until this study.

### 2.5. Physical Profile of Mud Volcanoes

**Figure 1:**
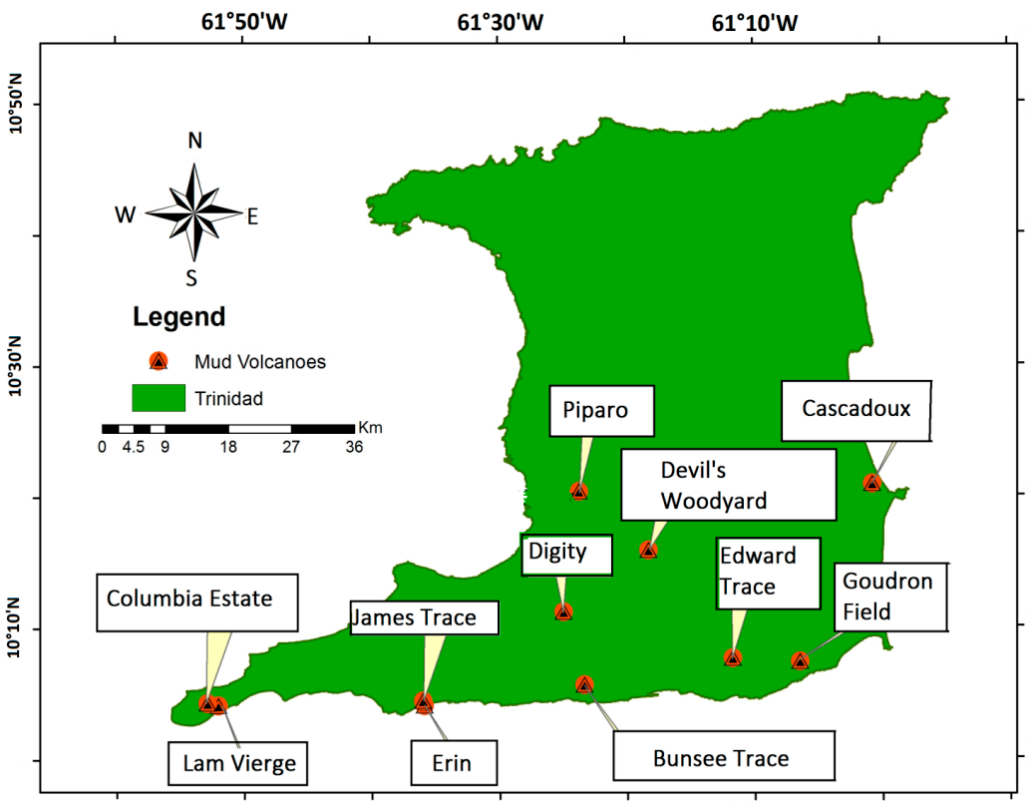
Map of Trinidad showing Mud Volcano Sampling Locations.

Eleven mud volcanoes were selected from 27 known mud volcanoes on the island of Trinidad [25]. Table 1 lists the sites with their respective GPS coordinates with descriptive notes and the locations are shown in Figure 1. Mud samples were collected from approximately 10 cm beneath the mud volcano crater surface.

**Figure 2:**
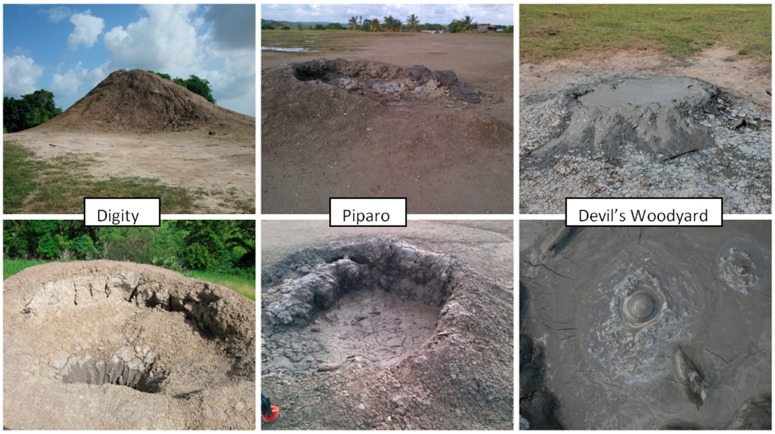
Selected mud volcanoes in Trinidad with representative cones.

In the Figure 2 below, three selected mud volcanoes sites (i) Digity (ii) Piparo and (iii) Devil’s Woodyard are shown with the close up of the vents below. In the vent of one of the cones of the Devil’s Woodyard, the bubbling gas is evident.

**Table 1:** GPS Locations and descriptions of Mud Volcano Sampling Sites.

Mud Volcanic Sites with Coordinates	Notes
Piparo N 10°20'20.7" W 061°23'43.1"	An active volcano in the island erupting in 1997 covering an area of 2.5 km^2^. The three major cones are less than 2 m tall. All over the site there are minute vents that show presence of gases escaping.
Devil’s Woodyard N 10°15'51" W 061°18'18.2"	Active in the past, the volcanic cones that it has generated are less than 30 cm in height and the openings at the volcano are on average 30 cm in diameter with evidence of small amounts of gas escaping.
Erin N 10°04'05.5" W 061°35'52.1"	This volcano is located at a height of 103 m above sea level. This volcano has been active recently and last erupted violently in the early 20th century.
Goudron Field N 10°07'20.0" W 061°06'28.4"	This is a field of volcanoes that is located in the oilfields of Guayaguayare and is one of the most populous in terms of the number of volcanoes. This field has no volcanic cones but very impressive ponds of very active bubbling mud. The bubbles are sometimes 60 cm in diameter.
Edward Trace N 10°07'37.1" W 061°11'43.5"	A spectacular field that is located in the forest of Moruga. There are many volcanic vents that show evidence of activity and the volcanic cones are growing as time passes. The volcanic cones have grown as high as 6 m in some cases.
Digity N 10°11'10.3" W 061°24'57.3"	This is by far the largest of the volcanoes considered with a height of 4 m. It consistently shows very little activity and gaseous escape. There is only one vent at the site.
Bunsee Trace N 10°04'6.9" W 061°52'01.7"	This mud volcano is located in Penal and is closer to the sea. This volcanic field has three main vents that are very active. There are a combination of cones and a thick viscous mud pond. It is active and is sparsely occupied by vegetation. The main vent is like a mud pond and there is a cone about 2 m high about 50 m away.
Cascadoux Trace N 10°20'52.1" W 061°00'44.9"	This mud volcanic cone is less than 2 m tall and shows very little evidence of activity. It is located in the East of Trinidad in the Ortoire district.
Lam Vierge N 10°04'06.9" W 061°52'01.7"	This is located in the Cedros district and is fairly unimpressive. It is located close to an oil field and stands less than 30 cm tall. Its ejecta is thick and show evidence of very little volcanic activity.
Columbia Estate N 10°04'16.8" W 061°52'52.1"	Located in the village Fullerton on the furthest part of the Southwestern peninsula, it has two vents showing signs of activity and has erupted very recently. The volcanic vents are very small and stand less than 60 cm..
James Trace N 10°04'26.1" W 061°35'59.7"	A field of easily accessible volcanoes that has a very unique feature about it. Unlike other mud volcanoes in this group that are being considered, some of the mud volcanoes here are surrounded by plants and vegetation. These volcanoes show evidence of fair amount of activity. They do not form cones but are small ponds of mud.

**Figure 3:**
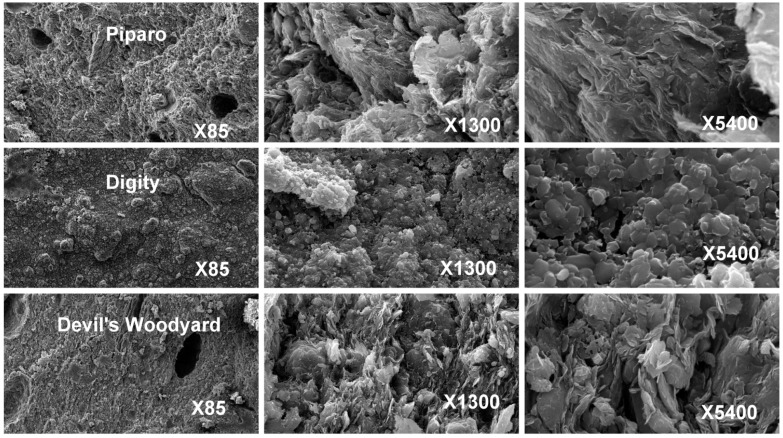
Microscopic features of dried mud volcanic samples from Piparo, Digity and Devil’s Woodyard at three magnifications.

The dried mud volcanic slurry from these three sites was subjected to electron microscopy to examine their structure at the microscopic level. This is shown in Figure 3.

The Devil’s Woodyard samples are seen to have formations of small globular structures, interspaced with voids. In the Digity sample, the globular structure is less defined, seemingly fused together so that the ball-like shapes are less apparent. It also appears less porous since there are fewer voids. The Piparo samples have even fewer voids still, and instead of small, well defined globules, they seem to be structured as larger clumps. Piparo and Devil’s Woodyard show evidence of flaky structure at the smallest scales.

Typically, the samples from the three different sites are reasonably uniform.

### 2.6. Elemental and Mineralogical Analyses

The elemental analysis used in detection and quantification of the elements was performed using X-ray fluorometry. For the elemental analyses, the eleven samples from the individual mud volcanoes were oven-dried to constant weight at 80 °C for 48 h. The pulverized samples were finely ground and compressed to form circular disc pellets. The pellets were fixed into designated holding cells and introduced into the Bruker-AXS Wavelength Dispersive X-Ray Fluorescence Spectrometer (WD-XRF, Madison, Wisconsin, USA) for testing. The carbon and sulphur determination was performed separately and more accurately using an ELTRA CS 2000 Carbon/Sulphur Analyser (Haan, Germany). Five (5) grams of the sample was placed into a crucible and introduced into the instrument to be combusted. The sulphur content was determined automatically by calculating the quantity of sulphur oxides generated and carbon with the quantity of carbon dioxide generated by complete combustion.

For the mineralogical analysis by X-ray diffraction (XRD) the eleven samples were oven-dried to constant weight at 80 °C for 48 h. The samples were finely ground and placed compacted in a circular disc shape in sample holder, for scanning. The settings for scanning were a full scan at 0.5° intervals, for 180°. The XRD analysis was carried out using a Bruker-AXS Diffrac Plus Spectrometer (Madison, WI, USA).

### 2.7. Microbial Life

The microbiological enumeration procedure was done using a standard spread plate technique. Sterile 1.3 m PVC (polyvinyl chloride) pipes were used to extract sediment samples. Samples were obtained from a 0.1 m depth. The microbes were cultured from 10^−1^, 10^−2^ and 10^−3^ dilutions. Triplicate aliquots (0.1 mL) of each dilution was spread on the surface of an agar plate and the plates were then incubated at 30 °C in a carbon dioxide environment. The cultures were incubated for one week after which the numbers of anaerobic colonies were counted.

### 2.8. Soil Property Experimental Methods

The methods used for the determination of the soil properties of water content, CEC and pH were standard procedures for soil property measurements [36]. The CEC procedure enabled the measurement of the CEC of a soil at its “field value” pH [37]. The method presented is a modification of a procedure called the unbuffered salt extraction by Grove [38]. Determination of percentage water content was done using the thermogravimetric method.

### 2.9. Methane Gas Analyses

Methane samples were collected from the mud volcanoes using Tedlar bags and returned to the laboratory for analysis. Methane analysis was carried out via gas chromatography using a Varian CP 3800 gas chromatograph with thermal conductivity detection. Quantization was achieved using external calibration with a methane standard (Sigma-Aldrich, St. Louis, MO, USA).

## 3. Results and Discussion

The eleven mud volcanoes of Trinidad were subjected to analyses to measure mud volcano chemical parameters. The data parameters are as follows:

(1) Mineralogy
(2) Elemental Analysis
(3) Soil Properties
(4) Methane Gas Concentrations

**Figure 4:**
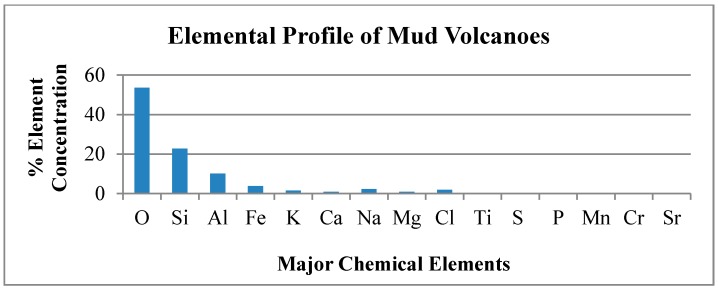
Average Elemental Concentration in the 11 Mud Volcanoes.

The average elemental analyses for the 11 mud volcanoes is depicted in Figure 4 below.

**Figure 5:**
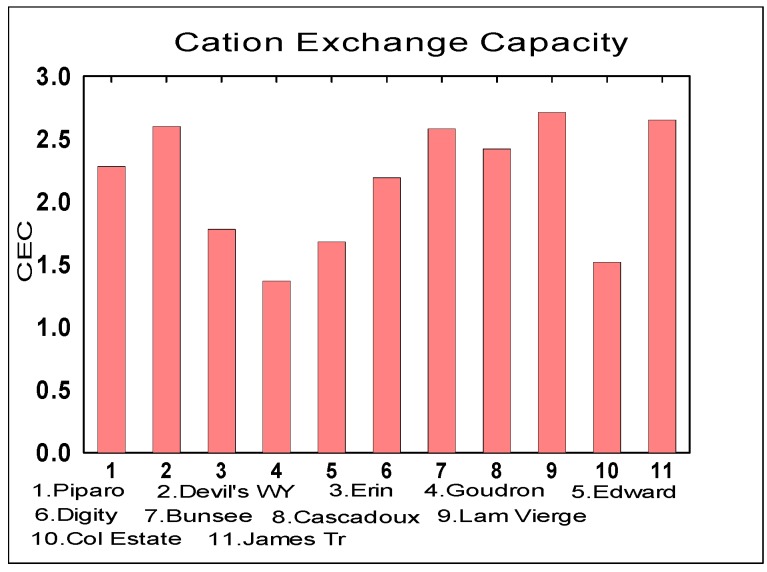
Cation Exchange Capacity (CEC) Comparison in Volcanoes (Mean = 2.16 ± 0.47).

**Figure 6:**
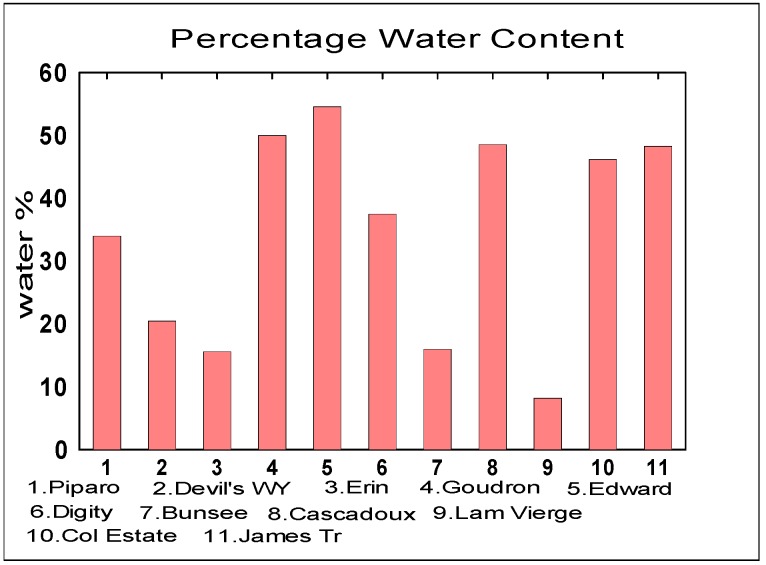
Percentage Water Content in Mud Volcanoes (Mean = 34.51% ± 15.85%).

**Figure 7:**
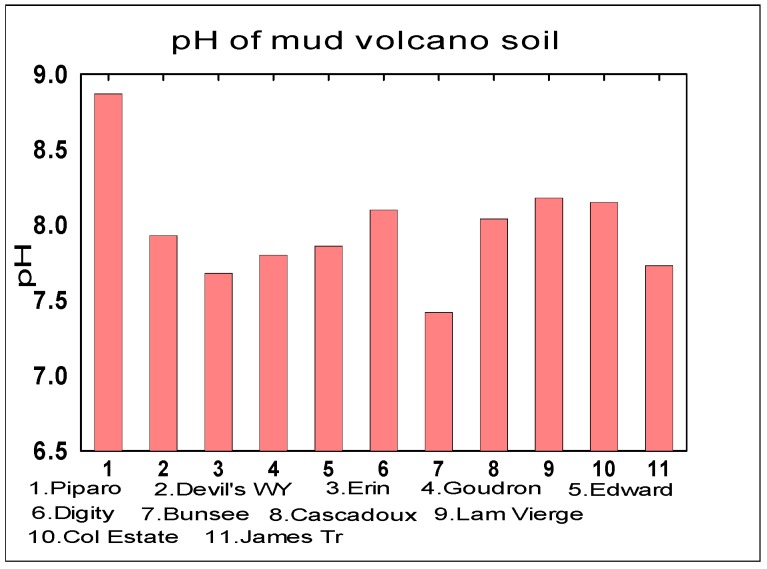
Comparison of Mud Volcanoes pH (Mean = 7.98 ± 0.37).

The soil properties measured were CEC, Percentage Water Content and the soil pH and Figure 5, Figure 6 and Figure 7 below respectively represent the findings.

The CEC measures the ability of a soil to exchange chemical cation species within the soil. The unit of CEC is kg.mol^−1^.

**Figure 8:**
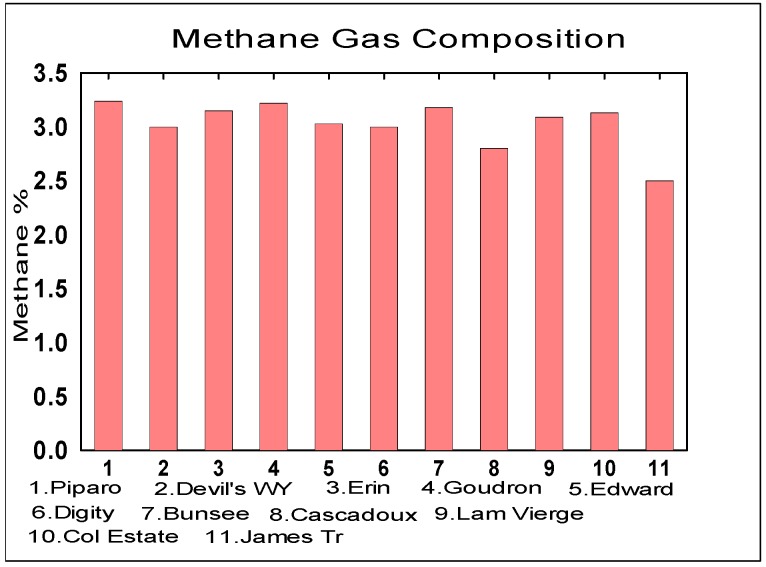
Mud Volcanic Methane Gas Compositions (Mean = 3.03% ± 0.22%).

The spectra for methane from all the mud volcanoes were consistent. Figure 8 shows the results.

**Figure 9:**
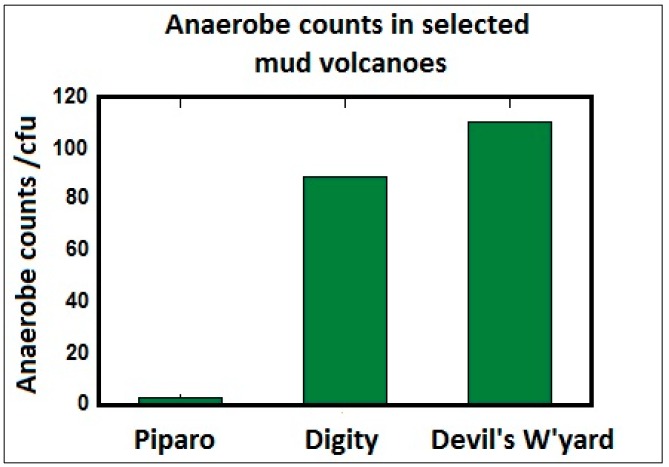
Microorganisms cultured under anaerobic conditions from selected mud volcanoes.

Figure 9 below shows the cultured microorganisms under anaerobic conditions, determined from plating technique normalized to 10^−1^ serial dilution which yielded mean anaerobic counts of 67 (cfu/mL).

Terrestrial mud volcanoes harbor microorganisms under anoxic conditions, limited nutrients and no light. These organisms thrive within the mud of the subsurface mud volcanoes. It was observed that the parameters of water content and pH parameters were within normal range, expected for microorganisms living in the mud at ambient temperature. However the anaerobes were living in a high carbon dioxide environment and extracting energy using inorganic chemical oxidation which may classify these organisms as lithoautotroph extremophiles. The chromium concentrations in the mud volcano show anaerobes that are metallotolerant. They seem also to be living in environments that are nutrient poor and could belong to the oligotrophic extremophiles category. These adaptations show the anaerobic organisms function as polyextremophiles although they thrive at ambient temperatures and conditions. It is however impossible to say if they also can survive under high pressure such as those deep underground as piezophiles as the samples obtained were not deep enough to qualify for that.

The XRF technique identified and quantified the percentage composition of all the different possible elements that could exist in the mud sample. The elemental concentration data was consistent for most of the mud volcanoes studied. The elemental concentration values of Devil’s Woodyard though, showed general significant positive and negative deviations from elemental mean values and was classified as an elemental outlier on this basis. Devil’s Woodyard shows a high sodium chloride content that exceeds the other volcanoes. Mineral interferences such as salt and deep crystalline rock deposits or influence by seawater in the underground system could account for this increased sodium and chlorine values [39].

Below in Table 2 we present the elemental composition of the Trinidad mud volcanic soils tested in this study.

**Table 2:** Elemental composition of the Trinidad mud volcanoes.

Chemical Element	Percentage Composition in Mud Volcanoes
O	53.60
Si	22.78
Al	10.10
Fe	3.72
K	1.55
Ca	0.90
Na	2.20
Mg	0.93
Cl	1.89
Ti	0.38
S	0.22
P	0.10
Mn	0.042
Cr	0.01
Sr	0.005

From Table 2, we note the elements most similar in percentage composition to Martian soil and those showing greatest deviation. The percentage composition of Silicon in the Trinidad mud volcanic soil is similar to the percentage composition of Silicon on Mars. Silicon in the Martian soil is fairly consistent between 18%–22% as determined by the Gamma Ray Spectrometer (GRS) on board the 2001 Mars Odyssey Mission for ±45° latitudes [40]. Silicon showed very little variation over the planetary surface ranging from 19%–22%, with the highest depletion at 18% near the volcanic regions of Tharsis Montes and Olympus Mons. There appears to be no evidence of globally distributed thick dust deposits of uniform composition and while the Silicon percentage in the Trinidad mud volcanoes is a little higher, it is still comparable for its importance as an analog site.

The element Chromium shows the highest deviation when comparing Mars soil conditions to the Trinidad mud volcanoes. The concentration of chromium in soil on Mars as determined by the Alpha Particle X-ray spectrometer on the Opportunity Rover in the region of Meridiani Planum is 0.46% ± 0.02% comparable to findings at Gusev crater and 0.20% in Bounce Rock samples [41]. The element showing the next extreme deviation is Strontium, which has the lowest concentration in the Martian soil, compared to the Trinidad mud volcanoes, as measured by the Mars Science Laboratory (MSL) rover Curiosity in the Gale Crater region with an average of 100 ppm, The Trinidad mud volcanic soil is ten times greater by percentage composition. We will discuss the implications of the role in biochemistry of microbial life of these three elements, Chromium, Strontium and Silicon as they represent the extreme deviations and greatest similarity respectively of the composition of the elemental percentages on the Martian environment and the mud volcano in Trinidad.

### 3.1. Mineralogical Analysis

The XRD analysis showed a consistent mineralogical character of the mud volcanoes studied. The sample spectra of the eleven volcanoes were compared with that of the known Devil’s Woodyard spectra performed by Knight [33]. The XRD spectra results for all selected volcanoes were consistent with Devil’s Woodyard with the mineralogy of 56% SiO_2_, 18% Al_2_O_3_, 7% Fe_2_O_3_ quartzite cemented sandstones calcite species siderite (FeCO_3_) conglomeratic baked clay and lignitic organics [33]. The results signify formation under similar conditions from the same parent material. The evidence also gives credibility to the assumption of volcanic system being a separate process with negligible mineralogical contributions from surrounding soil.

### 3.2. Soil Properties

#### 3.2.1. pH

The pH of the soils were consistent with mean pH values that were slightly alkaline 7.5–8.87 with maximum pH 8.87 at Piparo and lowest pH 7.42 at Bunsee Trace with an overall average of 7.98. The mean pH values are consistent with the literature values which are approximately ~7.5 for Martian soils [35]. These pH values also compare well with those found at the Phoenix lander site on Mars which were moderately alkaline of 7.7 ± 0.5 [42].

#### 3.2.2. Water Content

The percentage water content values from the different mud volcanic locations vary widely between from 8%–60% with Edward at the highest and Lam Vierge, as the lowest percentage water concentration. Percentage water variation may be due to groundwater influences beneath subsurface Trinidad. Groundwater influences entering at different locations into the mud volcanic reservoir at different places is feasible. Possible contamination is possible from spring seawater vents connected to some volcanoes. Marine bacteria found at the mud volcanic vent of Devil’s Woodyard is consistent with anaerobic methanotrophic marine bacteria being present in high saline environments [43,44]. Water plays a predominant role in soil texture. The water component is high and the particles of soil are fine, with no soil profiles. High water content in the mud gives the appearance of slurry. However a Mars–Earth comparison is difficult as there is no conclusive evidence of liquid water on the Martian surface, however one can speculate that it may have played a part in the past by the morphological evidence.

#### 3.2.3. Cation Exchange Capacity

The soil CEC of the mud volcanoes had an average value of 2.16 mol·kg^−1^. These CEC values are very low compared to the typical Trinidad soils which can reach as high as 40 [45]. The soil diversity of Trinidad is high and mud volcano ejecta is different from surrounding top soil. Low CEC values (<4) indicate that the valuable nutrients are easily leached from the soil. The mud volcano ejecta material cannot be classified as a soil but rather slurry due to its high water content and homogeneity. CEC comparisons between Earth and Mars are also impossible given the lack of obvious liquid water on Mars while it is necessary to understand the mud volcanic environment for these extremophiles on Earth.

### 3.3. Methane Gas Measurements

Methane gas production was used as an indicator of the metabolic activity in the mud volcano if it is indeed biogenic in origin from methanogens. The percentage by volume of the methane concentration at the different sites is fairly uniform. The mean value of the methane gas concentration of the eleven volcanoes is measured at 3.03% ± 0.22%. Atmospheric methane concentration ranges from 1.77–1.78 ppm on Earth compared to reports of up to 15 ppbv global average abundance in the Martian atmosphere found by Mars Express (MEX) [46,47]. It is noted that the origin of the Martian methane is still unclear as to whether it is biogenic or geochemical.

### 3.4. Silicon in Soil

Silicon exists as silicates and is responsible for making up bulk of clays in the soil. Clay soils have fine particles compared to different particles such as sand and have a much larger surface area to retain and supply nutrients such as calcium, magnesium, phosphate and water for plant uptake. Different silicate arrangement of atoms in clays gives rise to varying mineral arrangements such as vermiculite, montromollinite and kaolinite.

The soil is composed of high amounts of water and minerals contained in slurry. The mineralogy content of the mud volcano is consistent throughout the entire distribution of the southern part of Trinidad. Given that the mineralogical composition consists of clays kaolinite, montromollinite and vermiculite one can attribute the predominant silicon content to these factors [33]. Silicon binds with oxygen to form a tetrahedral lattice SiO_4_ which is the standard unit that polymerizes to create the different clay minerals that are present in the mud volcanic soil.

The role of silicates in the soil provides bulk and is responsible for the lattice structure of the clays. Silicon is involved in chemical reactions in the soil and surface area facilitates an environment for microorganisms but there is no evidence that it is actually involved in the microbial metabolism reactions of microorganisms in the biosphere. Organisms using silicon as part of their activities that incorporate them within their structure are well known. Marine diatomic organisms incorporate the silicon within the cell wall by micromorphogenesis, and used to describe the small-scale processes of silicification in the diatom, including the polymerization and formation of non-membrane bound nanostructures [48]. It is noted that the average percentage of silicon in the Martian soil is similar to the percentage in the mud volcano habitats in Trinidad. This is significant as it plays a part in forming the matrix in which the microbes can use as a habitat here on Earth and should they exist at all on Mars.

### 3.5. Chromium in Soil

There is much higher concentration of chromium on the Martian soil than the Trinidad mud volcanic samples as seen from Table 2. This value is almost forty times that of the concentration of chromium in the mud volcanoes of Trinidad which is 0.01% and can have implications for the Chromium tolerance of microbial life. Microbial communities were cultivatable in soil samples from Ivano-Frankivsk, Ukraine, with Chromium ranging from uncontaminated levels of 67 mg per kg of dry soil to contaminated levels of 5490 mg per kg of dry soil [49]. This study showed that while colony forming units of bacteria reduced to approximately half at highest levels of Chromium contamination, microbial life did survive when there was an increased concentration of Chromium by approximately eighty times in the soil samples. Therefore, the presence of microorganisms on Mars is feasible at higher concentrations of Chromium than found in the mud volcanoes in Trinidad which can be considered at uncontaminated levels. Chromium is represented with two different oxidation states in soil, but can exist in valences from −2 to +6. It is present in the environment mainly in the trivalent or hexavalent state. Trivalent Chromium is considered non-toxic to life. The toxicity of the chromium in the hexavalent state is 100 times more than that of the trivalent state of chromium. This means that in order to sustain life on Mars the bacteria living there must be extremely metallotolerant if chromium must exist in the hexavalent form on Mars. Therefore there must exist the possibility of a polyextremophile that under anaerobic conditions must be lithautotropic, oligotrophic and extremely metallotolerant to approximate the conditions of the mud volcano microbes. Extremophiles in the mud volcanoes are present with different adaptive metabolic strategies to survive in these environments.

The reduction of hexavalent chromium by organic carbon and soil catalyzed reactions, microorganisms such as Arthrobacter bacteria can reduce the toxic hexavalent chromium to the non-toxic trivalent chromium and use it as an energy source. Chromium reducing bacteria belong to a variety of genre such as Achromobacter Aeromonas, Agrobacterium, Bacillus, Desulphovibrio, Enterobacter, Escherichia, Micrococcus and Pseudonomas.

Arthrobacter species known as the A. Globiformis and A. Nicotianae, exhibit anaerobic metabolism [50]. The exact microbial profile in the mud volcanic soils of Trinidad is unknown.

Arthrobacter use electrons from the reduction of hexavalent chromium to trivalent as an energy source. It is probable that the mud ejecta are toxic for life with the exception of microbes specifically adapted to the mud matrix conditions. It is uncertain how oxidation states of chromate are distributed in the mud volcano. It is unlikely that the chromium exists only in a trivalent state and in fact exists also in a hexavalent state.

Chromium reducing bacteria require widespread high cell densities for significant Cr(VI) reduction to occur. Chromium reducing bacteria utilize a variety of organic compounds as electron donors for chromium reduction.

Bacterial reduction of Cr(VI) occurs aerobically and anaerobically. Organisms may also reduce Cr(VI) under anaerobic conditions via the mediation of either a soluble reductase, a membrane-bound reductase, or both, with the possible involvement of cytochrome b, c and d. The aerobic activity of Cr(VI) reduction is generally associated with soluble proteins which NADH as an electron donor required to drive the reaction or to provide enhanced activity [51,52,53]. In the absence of added electron donors, chromium-reducing organisms utilize endogenous reserves for the reduction of Cr(VI) through the activity of soluble reductase [52,53,54]. Under anaerobic conditions Cr(VI) serves as a terminal electron acceptor through the respiratory chains of E. Cloacae, *E. Coli* and D. Vulgaris [52,55,56]. Chromium reducing bacteria are wide spread but only two species A. Radiobacter EPS-916 41 and *E. Coli* ATCC are known to reduce Cr(VI) in liquid media aerobically and anaerobically. These organisms reduced Cr(VI) better under anaerobic conditions than under aerobic conditions. A. Radiobacter EPS-916 actively reduced 0.05 mM chromate while growing aerobically but reduced up to 0.15 mM chromate under anaerobic conditions [57,58].

### 3.6. Strontium in Soil

Strontium was the element which was found to be in the highest percentage compostion in the Trinidad mud volcanoes compared to the Martian values of Strontium, from the Mars Science Laboratory rover Curiosity [59]. The strontium ion (Sr^2+^) is very similar to the calcium ion (Ca^2+^) both chemically and physiologically and it can substitute for calcium in physiological processes. Substitution of strontium for calcium in metabolic experiments can occur but results in kinetically slower reactions compared to similar reactions of calcium. When strontium is consumed by microorganisms they are metabolized in very much the same way as calcium. Strontium substituting for calcium in metabolic reactions that normally require calcium ions (Ca^2+^) or magnesium ions (Mg^2+^), in calcium deficient environments on Mars could feasibly support life but the mud volcanoes as an analog to the Martian environment is less of a match with regard to this element.

The exact identities and therefore the exact nutritional strategies of the bacteria in the Trinidad mud volcanoes are not known. Ali et al found that the microbes in the Digity, Piparo and Devil’s Woodyard were anaerobic methanogenic organisms that produce methane [23]. We propose that they can be affected by the chemical environment of silicon, chromium and strontium in their metabolism. As an analog to the Martian environment, the silicon acts as the matrix for the microbial environment in both scenarios while the effect of chromium in Martian soil will be stronger than in the terrestrial mud volcanoes for microbial life and could reduce but not eliminate the presence of microbial life on Mars [49]. The effect of the low levels of strontium on Mars compared to the mud volcanoes of Trinidad can be offset by the presence of higher levels of magnesium and calcium on Mars which can substitute for it in similar metabolic processes. The lower levels of strontium therefore also do not preclude the presence of possible microbial life on Mars.

## 4. Conclusions

The mud volcanoes of Trinidad can serve as analogs for the Martian environment due to the presence of morphologically similar structures on Mars such as in Acidalia Planitia and the Arabia Terra regions. The eleven mud volcanoes studied were found to be active with the following features:

- All emit methane gas at a consistent 3% by volume.
- The average pH of the soil sample was 7.98.
- The average CEC was 2.16 kg/m.
- The water content showed most variability with an average water content of 34.5%.
- Oxygen and silicon were the most dominant elements in the soil with chromium and strontium at low percentage composition of 0.01% and 0.005% respectively.
- Percentage silicon by composition was 22% comparable to reported Martian values.
- Chromium and strontium showed the highest deviation compared to values on Mars and the Trinidad mud volcanoes.
- Bacterial colonies were cultured under anaerobic from soil samples from Digity, Piparo and Devil’s Woodyard.

The chemical and physical environment of the Trinidad mud volcanoes have supported the existence of polyextremophile microbes which are found to be lithoautotrophic, oligotropic and metallotolerant thriving under an anaerobic environment. This may be representative of the microbial community that could existin subsurface of Mars if there is such.
